# The HIV-1 Antisense Gene ASP: The New Kid on the Block

**DOI:** 10.3390/vaccines9050513

**Published:** 2021-05-17

**Authors:** Zahra Gholizadeh, Mohd. Shameel Iqbal, Rui Li, Fabio Romerio

**Affiliations:** Department of Molecular and Comparative Pathobiology, Johns Hopkins University School of Medicine, Baltimore, MD 21205, USA; zgholiz1@jhu.edu (Z.G.); miqbal9@jhmi.edu (M.S.I.); rli74@jhmi.edu (R.L.)

**Keywords:** HIV-1, natural antisense transcription, antisense protein (ASP), overprinting, replication, pathogenesis, spread

## Abstract

Viruses have developed incredibly creative ways of making a virtue out of necessity, including taking full advantage of their small genomes. Indeed, viruses often encode multiple proteins within the same genomic region by using two or more reading frames in both orientations through a process called overprinting. Complex retroviruses provide compelling examples of that. The human immunodeficiency virus type 1 (HIV-1) genome expresses sixteen proteins from nine genes that are encoded in the three positive-sense reading frames. In addition, the genome of some HIV-1 strains contains a tenth gene in one of the negative-sense reading frames. The so-called Antisense Protein (ASP) gene overlaps the HIV-1 Rev Response Element (RRE) and the envelope glycoprotein gene, and encodes a highly hydrophobic protein of ~190 amino acids. Despite being identified over thirty years ago, relatively few studies have investigated the role that ASP may play in the virus lifecycle, and its expression in vivo is still questioned. Here we review the current knowledge about ASP, and we discuss some of the many unanswered questions.

## 1. Introduction: De Novo Creation of Genes

**Figure 1:**
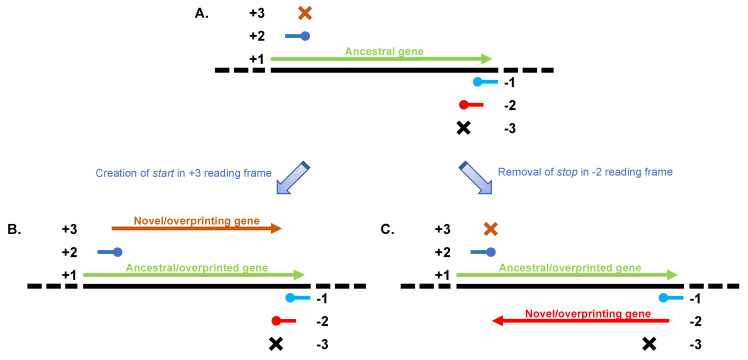
De novo creation of genes by overprinting. Typically, genomic regions contain a single gene encoding a protein product in one of the 6 reading frames (for example, reading frame +1 in panel (**A**). The same genomic region does not encode any protein in the other 5 reading frames, either because of absence of *start* codons (as in reading frame +3 and −3, marked with ×) or because of the presence of *stop*s shortly after a *start* codon (as in reading frames +2, −1 and −2, marked with ●). The occurrence of single point mutations in one of these 5 reading frames can give rise to a new protein-coding gene, either by creating a new *start* codon (as in reading frame +3 in panel (**B**) or by eliminating a *stop* codon (as in reading frame −2 in panel (**C**). When this occurs, the original gene is called ‘ancestral’ or ‘overprinted’, while the new one is called ‘novel’ or ‘overprinting’. In rare cases, both events can occur over time (as in the case of HTLV-1 and HIV-1), thus giving rise to genomic regions that contain three overlapping protein-coding genes.

In the majority of cases, new genes are created by transfer of existing genetic material [1]. This can occur in various forms: exon shuffling, gene duplication, retroposition, lateral gene transfer, and gene fusion or fission [1]. However, in rare cases, genes can also be created de novo. This mechanism was thought to be rare [2], but recent studies show that it occurs frequently. De novo gene creation can occur in intergenic regions and introns [3,4], but also in genomic regions that already contain a protein-coding gene—a process called ‘overprinting’ (Figure 1). In this case, a genomic region with an existing coding sequence in one of the six reading frames (Figure 1A) undergoes point mutations in one of the other five reading frames that generate a new *start* codon (Figure 1B) and/or remove *stop* codons (Figure 1C), giving rise to a second coding sequence. The resulting genomic region contains two overlapping open reading frames (ORFs): the ancestral or ‘overprinted’ gene, and the novel or ‘overprinting’ gene [5]. For most gene pairs, the identification of the ancestral and the de novo gene can be determined with high accuracy because the former presents a much wider phylogenetic distribution than the latter [6]. Indeed, proteins that are created de novo by overprinting do not have homologs in other organisms and are at times referred to as taxonomically restricted or ‘ORFans’ [7,8]. However, it is also possible that de novo proteins may be members of a family of proteins that have diverged beyond recognition or that were lost [9].

## 2. Overprinting in Viral Genomes

Overprinting has been documented in both prokaryotic and eukaryotic genomes [10,11,12,13,14,15,16,17]. However, the incidence of overlapping genes in these organisms is relatively low [18,19,20]. On the other hand, overprinting is quite frequent in viral genomes [21,22]. Indeed, the first evidence of overlapping genes came from the bacteriophage ΦX174 [23], then followed by many examples in several eukaryotic viruses. Two theories have been proposed to explain the high abundance of overlapping genes in viral genomes [24]. The gene-compression theory states that error-prone viral polymerases and biophysical constraints imposed by the viral capsid drive the creation of overlapping genes that allow maximization of the amount of information that can be encoded in small genomes [25]. The gene-novelty theory asserts that de novo creation of genes is driven by selective pressure, and gives rise to gene products that provide a selective advantage to the virus, and thus become fixed in the population [5].

The high abundance of overlapping genes in viral genomes has allowed the use of statistical and computational methods to investigate the composition bias, structural features, evolution, and potential function of overlapping gene pairs and de novo proteins [5,24,26,27]. Sequence composition varies greatly between overlapping and non-overlapping genes, as well as between ancestral and novel genes in overlapping pairs [27]. Pavesi et al. showed that, in the case of overlapping pairs, the codon usage of ancestral genes is more similar to the rest of the genome than that of novel genes [26]. While this difference decreases with the age of de novo genes [6], constraints imposed by the ancestral gene might prevent the novel gene from acquiring a codon usage completely similar to that of the rest of the genome [26]. Moreover, young novel genes initially evolve rapidly under positive, or weakly purifying selection, while older novel genes evolve more slowly under increasingly stronger negative selection [6]. Interestingly, in some cases overlapping genes show ‘asymmetric evolution’ whereby the two members of a pair evolve at different rates [28]. Analysis of composition bias of proteins encoded by overlapping genes found that they are enriched in high-degeneracy amino acids (arginine, leucine, serine), which may alleviate evolutionary constraints acting on the pair of overlapping genes at the DNA level [27,29]. In addition, proteins encoded by overlapping genes are enriched in amino acids with high propensity toward structural disorder (arginine, proline, serine) [5,27,30,31]. This is an essential state of many proteins that, in the absence of a binding partner, lack a stable secondary and tertiary structure, and adopt a number of rapidly interconverting structural forms rather than a particular three-dimensional structure. Structural disorder affords proteins encoded by overlapping genes greater freedom of sequence evolution without loss of function [5]. Indeed, proteins with high intrinsic structural disorder are more likely to tolerate amino acid substitutions that maintain disorder.

The creation of a novel ORF by overprinting and its subsequent fixation in the viral population strongly suggest that the new protein provides a selective advantage [24]. Although most de novo genes encode for accessory proteins, they appear to play a role in the pathogenicity or spread of the virus [5]. Studies have shown that de novo proteins act by neutralizing the interferon and RNA interference responses of the host [32,33,34,35], by inducing apoptosis [36,37], or by promoting systemic spread of the virus [38]. Therefore, there is evidence that creation of novel genes by overprinting allows viruses to make virtue out of necessity.

## 3. A Special Kind of Overprinting: Antisense Genes

Most cases of overlapping genes studied to date are encoded in two different reading frames of the same DNA strand. There is also evidence of overlaps that involve three different ORFs. This is observed, for instance, in two human retroviruses: HIV-1 and HTLV-1. For HIV-1, the overlap involves the *env*, *tat*, and *rev* genes [39], and for HTLV-1 it involves the *p13/p30*, *tax*, and *rex* genes [40]. Significantly fewer are the examples in which the pair of overlapping ORFs are encoded on different DNA strands, and therefore in opposite orientations.

**Figure 2:**
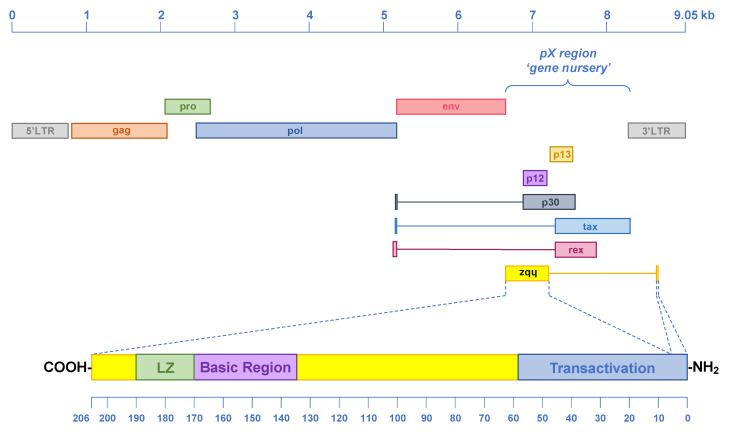
Genomic organization of the HTLV-1 provirus and main structural features of HBZ. The *hbz* gene is located within the *pX* region of the proviral genome gene, and it overlaps the p12 and p30 ORFs. HBZ is a basic leucine zipper (bZIP) transcription factor of 206 aa, and it contains three main domains: the transactivation domain, the basic region, and the leucine zipper (LZ). HBZ is involved in the establishment of HTLV-1 latency by preventing the recruitment of the HTLV-1 transactivator Tax to the proviral 5′LTR region.

Without a doubt the viral antisense gene most intensely studied is the HTLV-1 basic leucine zipper (bZIP) factor (*hbz*) gene [41] (Figure 2). This gene maps in the pX region of the HTLV-1 genome, which has been called a ‘gene nursery’ due to the presence of high number of new genes and overprinting events [26]. The *hbz* gene is expressed via both spliced and unspliced antisense transcripts that originate from the proviral 3′LTR, which has been shown to contain a bidirectional promoter [42,43]. The negative sense promoter in the HTLV-1 3′LTR lacks a TATA box, relies on Sp1 binding sites, and is not under the control of the HTLV-1 transactivator TAX protein [43,44]. The *hbz* gene encodes for a 206-aa protein (HBZ) that plays a key role in the establishment of HTLV-1 chronic infection. Indeed, HBZ appears to promote proviral latency via interaction with several host factors that bind the viral cyclic AMP Response Elements (vCRE) in the HTLV-1 5′LTR, such as CREB, CREM and ATF-1 [41]. Interaction of HBZ with these factors precludes recruitment of TAX to the 5′LTR, and ultimately downregulates sense transcription [45]. Additionally, HBZ has been shown to prevent the binding of TAX to the host factor CBP/p300, and its recruitment of the 5′LTR [41]. There is evidence that HBZ may play a role in the leukemic process following HTLV-1 infection. Indeed, in about half of the ATL cases, the *tax* gene is not expressed due to deletion of the 5′LTR [46], epigenetic silencing of the 5′LTR [47], or mutations within the *tax* gene [48]. On the contrary, the 3′LTR and the pX region of HTLV-1 remain intact, and *hbz* is transcribed in all ATL cases [42]. In addition, knockdown of HBZ inhibits proliferation of ATL cells [49]. Finally, expression of HBZ in transgenic mice was shown to cause T cell lymphomas and inflammatory diseases that do not appear in TAX transgenic mice [50,51]. Altogether, there is strong evidence for the implication of the HTLV-1 antisense protein HBZ in leukemogenesis.

Antisense genes are also found in other human and animal retroviruses. HTLV-2, 3 and 4 express the antisense proteins, APH-2, 3 and 4, respectively [52]. These proteins are similar to HBZ, suggesting that these ORFs were created in the common ancestor of HTLV-1/4 after it diverged from bovine leukemia virus (BLV) [26]. The simian T-cell leukemia virus (STLV) expresses the simian bZIP (SBZ) protein with function similar to HBZ [53]. An antisense ORF is present in the feline immunodeficiency virus (FIV) genome. However, while FIV is capable of producing antisense transcripts, expression of an antisense protein has not been detected [54]. Similarly, murine leukemia virus (MLV), bovine immunodeficiency virus (BIV) and BLV are capable of antisense transcription, but expression of antisense proteins by these viruses has not been demonstrated [52].

## 4. The HIV-1 Antisense Protein, ASP

**Figure 3:**
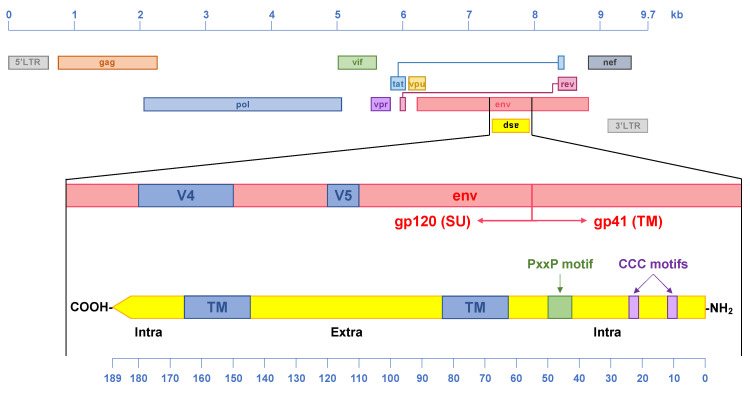
Genomic organization of the HIV-1 provirus (group M) and main structural features of ASP. The *asp* gene is located within the *env* gene, and it straddles the gp120 (SU) and gp41 (TM) boundary. In particular, the *asp* gene overlaps the *env* gene in the region encoding the V4 and V5 loops of gp120. ASP encodes a protein of ~189 aa (the actual length varies across HIV-1 clades). It is predicted to contain two transmembrane domains (TM, between residues 63–84 and 146–167). The N-terminus and C-terminus are intracellular, while the portion between the two TM domains is extracellular. The N-terminus also includes highly conserved motifs: two cysteine-triplets, and a potential SH3 domain-binding PxxP motif.

The existence of an antisense gene in the HIV-1 proviral genome was first proposed more than 30 years ago with the identification of a highly conserved ORF in the minus strand of twelve viral isolates [55]. The *asp* ORF, located in the same genomic region as the *env* gene straddling the gp120/gp41 boundary (Figure 3), was also present in an additional 12/13 *env* sequences available (i.e., incomplete genome sequences) [55]. This newly identified HIV-1 gene was predicted to encode for an antisense protein (ASP) of ~190 residues, rich in hydrophobic amino acids and possibly associated with cellular membranes [55]. The hypothesis that the newly identified ORF might encode for a real protein was based on three lines of evidence. First, the ASP ORF is >300 nucleotides (i.e., >100 codons), which is uncommon in DNA strands complementary to known genes [56]. Second, the signal sequences necessary for production of an antisense mRNA transcript (promoter, poly-A addition signal, poly-A addition site, downstream G and T domains) are present and conserved in all sequences analyzed. Third, sequences necessary for the translation of a protein (canonical *start* and *stop* codons, a codon periodicity of ‘G-nonG-N’) are also present and conserved. Altogether, these analyses provided convincing theoretical evidence for the presence of a protein-coding antisense gene in the HIV-1 proviral genome [55].

At a glance, ASP presents several conserved features that may have functional significance (Figure 3). Although the crystal structure of ASP has not been resolved, sequence analysis and computer modeling suggest that ASP possesses two transmembrane (TM) domains comprised approximately between residues 65–85 and 145–165. The portion of the molecule between the two TM domains constitutes an extracellular loop, while the N-terminal and C-terminal ends of ASP are intracellular [57,58]. Additional features of interest are present in the N-terminal domain. They include, two closely spaced cysteine triplets located in the first 25 residues of the protein. Their function has not been investigated, but they might form disulfide bonds that stabilize the protein or coordinate heavy metals such as Zn^2+^ and Ni^2+^. In addition, the N terminus of ASP includes a highly conserved PxxPxxP motif located between residues 40–50 of the protein. This motif is reminiscent of Src homology 3 (SH3) domain-binding motifs found in many proteins, including HIV-1 Nef [59]. Biochemical and functional studies are needed to establish whether the PxxP motif of ASP is a bona fide SH3 domain-binding motif. Finally, the intracellular portion of the C-terminal domain of ASP does not seem to contain conserved domains or motifs. This is in part due to the fact that it overlaps the V4 loop of gp120 on the opposite strand. Indeed, many HIV-1 strains and subtypes (including some of the ones with high prevalence, see below) express an ASP that is truncated shortly after the second TM domain, and that lacks the intracellular C-terminal domain altogether, suggesting that it may be dispensable for full ASP function. A more thorough and systematic experimental analysis is needed to fully evaluate the functional role of these and possibly other yet unidentified ASP domains.

## 5. ASP Expression in HIV-1 Infected Individuals

At least two recent reports have documented expression of HIV-1 antisense transcripts in cells collected from donors on ART using RT-qPCR [60,61] (Table 1). Our lab used a strand-specific RT-qPCR assay to detect levels of antisense transcripts ranging from 10 to 30 copies per million resting CD4+ T cells [60]. Using a different approach, Mancarella et al. observed similar levels of antisense transcripts but only following ex vivo anti-CD3/CD28 stimulation of donor CD4+ T cells [61]. While direct evidence that ASP protein is expressed in HIV-1 infected individuals is still missing, multiple studies over two decades have documented the presence of humoral and cellular immune responses against ASP in HIV-1 infected individuals (Table 1). An early report by Vanhée-Brossollet et al. showed that sera obtained from 15 different HIV-1 infected patients, but not sera from HIV-1 negative individuals, was able to immunoprecipitate in vitro-translated ASP [62]. More recently, Savoret and colleagues demonstrated that antibodies to ASP are detectable in serum of HIV-1 patients who are off antiretroviral therapy [63].

A few studies also demonstrated the presence of CD8+ T cell responses against ASP-derived peptides in HIV-1 infected patients. Champiat et al. identified multiple alternate reading frames (ARFs) in both orientations of the HIV-1 genome, but most of them were present in the *env* region and in the same reading frame as ASP [62]. They then designed 9-mer peptide pools for each ARF, including 3 distinct pools based on the ASP ORF, and tested whether CD8+ T cells from HIV-1 infected individuals showed reactivity against these peptide pools. While no reactivity toward ASP-specific peptide pools was detected with cells from acutely-infected patients, CD8+ T cells from chronic patients (both on and off antiretroviral therapy) did react against ASP-derived peptides [62]. A subsequent study focusing exclusively on ASP detected CD8+ T cell responses against ASP in HIV-1 positive subjects off antiretroviral therapy [63]. In addition, this study found that CD8+ T cells from these patients produced multiple cytokines and chemokines when exposed to ASP-derived peptides. Similar results were published in an independent report that investigated CD8+ T cell responses against five different antisense ORFs in the HIV-1 genome, including ASP [64].

Taken together, these studies provide indirect evidence that ASP in expressed during the course of HIV-1 infection. However, this does not indicate that ASP is a protein that plays a role in the virus lifecycle.

## 6. Expression and Functional Role of ASP in In Vitro Models

**Figure 4:**
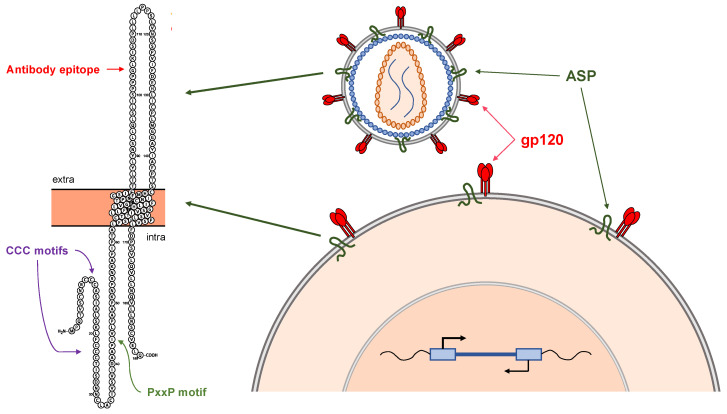
Association of ASP with the plasma membrane and the viral envelope. Studies from our and other groups have shown that ASP is expressed on the surface of productively infected cells. In addition, our studies demonstrate that ASP co-localizes with gp120 on the surface of primary HIV-infected CD4+ T cells and macrophages. A putative model of membrane-associated ASP is shown on the left side of the figure. According to this model, the N-terminus and C-terminus of ASP are intracellular (or intraviral), while the segment between the two transmembrane domains is exposed in the extracellular (or extra-viral) milieu. This model is supported by evidence that our monoclonal antibody directed against an epitope in the predicted extracellular loop of the protein (residues highlighted in red) can detect ASP on the cell surface in flow cytometry and confocal microscopy studies without the need from membrane permeabilization [57]. The same monoclonal antibody could detect ASP on the surface of viral particles (fluorescence correlation spectroscopy), and could capture HIV-1 virions (virion capture assay) [57].

Relatively few studies have investigated the expression of ASP in infected cells, and its role in HIV-1 infection. Exploring these research areas has been hindered by several challenges. First, ASP is expressed at very low levels. The negative sense promoter (NSP) located in the HIV-1 3′LTR lacks a TATA box, is Tat-independent, and relies primarily on ubiquitous, housekeeping transcription factors [67,68,69]. Therefore, NSP drives the expression of antisense RNA at low levels. In addition, HIV-1 antisense transcripts are not efficiently exported into the cytoplasm, possibly due to inefficient polyadenylation. All these factors contribute to ASP being expressed in low amounts. Second, reagents needed to study ASP expression are lacking. Antibodies directed against ASP are still not commercially available. In addition, ASP is poorly immunogenic, which has made it difficult to raise potent and specific antibodies. Recently, two anti-ASP monoclonal antibodies have been reported: one is directed against a 16-aa epitope located immediately before the first predicted transmembrane domain [70], the other directed against a 13-aa epitope located between the two predicted transmembrane domains in the extracellular loop of ASP (Figure 4) [57]. However, perhaps what makes ASP especially difficult to study is, at the same time, what makes it so special and peculiar: the fact that it is a gene created de novo, and that it is entirely encased within the *env* gene. Indeed, ASP does not have any known orthologs, paralogs or xenologs [71]. Therefore, we do not have any information from other viruses that might enlighten us about a possible role for ASP in HIV-1 replication, and guide us in developing hypotheses. In addition, functional studies involving mutagenesis of ASP (e.g., deletion or substitution of large sequences or domains) would also affect the *env* sequence on the opposite DNA strand of the genome. Thus, while ‘work-around’ solutions are possible, the genomic location of the ASP gene makes it quite challenging to study its function.

Despite these limitations, a few publications have reported the expression of ASP in various cellular systems, and have made progress in elucidating its (possible) role in HIV-1 infection. In an early study, Briquet and Vaquero studied the sub-cellular localization of ASP in multiple in vitro cell systems, namely A3.01 cells over-expressing a fusion VSV-ASP protein, chronically infected ACH-2 cells, and SupT1 transfected with a molecular clone expressing HIV-1_HXB2_ (mimicking acute infection) [72]. For detection of ASP, the authors used electron microscopy after immunostaining with anti-VSV antibodies (for VSV-ASP fusion protein), or rabbit antiserum directed against two N-terminal and one C-terminal peptides of ASP (for chronically infected ACH-2 and acutely infected SupT1 cells). In A3.01 cells over-expressing VSV-ASP, the authors found ASP associated with multiple membrane systems (plasma, mitochondrial and nuclear membranes). In chronically infected ACH-2 cells, ASP was expressed at very low levels in unstimulated cells, but after activation ASP became readily detectable both in the nucleus and in the cytoplasm. A similar expression pattern was visible also in acutely infected SupT1 cells [72]. The sub-cellular localization of ASP was also the focus of a later study from the Mesnard and Barbeau groups [58]. In their 2011 report, the authors used anti-FLAG antibodies in confocal microscopy to investigate ASP localization in Jurkat cells transfected with a construct expressing Flag-tagged ASP. These studies showed that ASP localized to the plasma membrane (with both polarized and unpolarized expression patterns) as well as with cell surface protrusion [58]. A subsequent report by the same groups confirmed these results in primary monocyte-derived macrophages infected with VSV-pseudotyped HIV-1 expressing Flag-tagged ASP [73]. More recently, our group reported a more in-depth analysis of ASP expression in several chronically infected lymphoid and myeloid cell lines and also in acutely infected primary human CD4+ T cells and monocyte-derived macrophages (MDM) [57]. For these studies, we developed a mouse monoclonal antibody directed against a peptide that maps in the putative extracellular domain of ASP (Figure 4). Flow cytometry and confocal microscopy studies showed that ASP localizes within the nucleus of all unstimulated, non-productively infected lymphoid and myeloid cell lines. Interestingly, ASP displayed a polarized nuclear distribution, and an accumulation in regions of the nucleus that contain actively transcribed chromatin [57]. Following cell stimulation and reactivation of productive infection, we found that ASP translocates to the cytoplasm, it associates with the plasma membrane (Figure 4), and thus becomes detectable with our monoclonal antibody without cell permeabilization, proving that this antibody recognizes an epitope exposed in the extracellular milieu. Further, our studies demonstrate that once present on the plasma membrane, a large fraction of ASP localizes in close proximity of the HIV-1 envelope glycoprotein gp120 (Figure 4). These results were confirmed by super resolution microscopy and also in acutely infected primary CD4+ T cells and MDM [57]. Expression of ASP in close proximity of gp120 on the plasma membrane suggested that ASP may be also present on the envelope of viral particles upon budding and release from infected cells, which we were able to demonstrate by both fluorescence correlation spectroscopy and virion capture assay (Figure 4) [57].

While the expression pattern and sub-cellular localization of ASP have been addressed in several studies that yielded largely consistent results, the role of ASP in viral replication has been explored to a much lesser degree. This is in part due to the lack of ASP homologs and the fact that *asp* and *env* are overprinting genes. To assess a potential role of ASP in viral replication, Clerc et al. substituted a single nucleotide within the twelfth codon of the ASP gene in the infectious HIV-1 molecular clone NL4-3 strain, which replaced a cysteine codon with a *stop* codon (TGC > TGA), thus resulting in the early termination of ASP [58]. At the same time, this substitution resulted in a synonymous mutation in *env* on the opposite strand. Infection of Jurkat cells with equal amounts of the wild type and ASP-deficient HIV-1_NL4-3_ did not show any difference in the replication dynamics of the two viruses over 14 days. However, this negative result could be due to various reasons. For example, as discussed above, our studies showed that ASP is present in the HIV-1 viral envelope and is exposed to the ‘extra-viral’ milieu [57]. This might suggest a possible accessory role of ASP during the early steps of HIV-1 infection (e.g., attachment, fusion, or entry). In the study by Clerc and colleagues that sought to evaluate differences in the replication rates of wildtype vs. ASP-deficient HIV-1, the use of a cell line model expressing high cell surface levels of CD4 and CXCR4 might have allowed optimal infection efficiency even in the absence of ASP. Moreover, T cell lines offer sustained transcription levels, which might contribute to minimize the effects caused by the loss of an ‘accessory’ gene. Therefore, it is possible that the experimental system may have contributed to hide small but appreciable differences in the replication efficiency between wildtype and ASP-deficient HIV-1.

Two studies from the Barbeau group provided evidence that ASP expression might be responsible for the induction of autophagy in infected cells. In the first report, the authors found that codon optimization improved ASP expression levels and facilitated its detection after transient transfection [74]. Further, this study showed that ASP forms high molecular weight aggregates that are stable even in the presence of detergents and reducing agents. More interestingly, this report demonstrated that overexpression of ASP induces autophagy, as shown by a punctate distribution of ASP reminiscent of autophagosomes, and by induction of autophagy markers LC3b-II and Beclin 1. Moreover, inhibition of autophagy with 3-methyladenine resulted in increased levels of ASP, suggesting that it may be degraded during the last step of the autophagy process [74]. In a more recent study, Liu et al. reported that ASP is ubiquitinated, and that it promotes autophagy by interacting with the host factor, p62 [70]. In addition, the authors demonstrated that ASP from all HIV-1 clades induces autophagy [70]. Both DNA and RNA viruses utilize autophagy to their advantage during their replication cycle [75]. In the case of HIV-1, autophagy is required for processing of GAG during infection of macrophages, and it significantly increases viral production [76].

These findings are very intriguing and point to two potential roles of ASP in HIV-1 replication. When present on the viral surface, ASP might be involved in virus entry, whereas when expressed within infected cells, it might promote autophagy. These roles are not mutually exclusive, because they occur at different stages of the virus life cycle. An early study did not show a reduction in viral replication when the ASP ORF was disrupted [58], but this may be due to limitations of the system that was used. Indeed, the role of ASP in HIV-1 replication may be small when observed in the limited setting of certain in vitro models that monitor a few cycles of viral replication, and yet significant when observed in the context of the infection in vivo and/or at the population level. Therefore, future studies will require a more careful evaluation of the model system used to determine whether ASP plays a role HIV-1 replication.

## 7. Origin, Conservation and Evolution of ASP

An important and still unanswered question that may help shed light onto the role, if any, that ASP plays in HIV-1 replication, spread and pathogenesis is ‘*where does ASP come from?*’.

This question was the focus of a 2016 study published by Cassan and colleagues [71]. Here, the authors analyzed ~23,000 HIV-1 (groups M, N, O and P), SIVcpz, and SIVgor *env* sequences obtained from ~3900 infected humans and nonhuman primates. These sequences were aligned to the reference HIV-1_HXB2_ sequence, which contains an ASP ORF of 189 codons between nucleotide positions 1717 and 1151 of the *env* gene in the −2 frame. To identify a similar ORF in the ~23,000 sequences being analyzed, the authors searched for the presence of an open reading frame of >150 codons (identified by canonical *start* and *stop* codons) located between the same two reference positions in *env* indicated above. Using these criteria, they found that 77% of the sequences from HIV-1 group M—which is responsible for the world pandemic—contain an ORF with a median length of 182 codons. When looking at high prevalence subtypes A, B, C, G and CRF01_AE (combined prevalence 81%) and low prevalence subtypes D, F, J, H and K (combined prevalence 3%), the ASP ORF was present in 84% of the former vs. 45% of the latter. Moreover, the ASP ORF was found in less than 1.5% of HIV-1 sequences that belong to non-pandemic HIV-1 groups N, O and P (combined prevalence ~0.1%). Finally, an ASP ORF as defined based on the criteria outlined above was absent in the sequences of SIVcpz and SIVgor that were analyzed. However, it is interesting to note that the median length of the ASP ORF in SIV becomes longer as the corresponding viral strain approaches phylogenetically to HIV-1 group M (66 codons for SIVcpz_Pts and 125 codons for SIVcpz_Ptt). Therefore, these studies showed that an ASP ORF of >150 aa is present almost exclusively in the pandemic HIV-1 group M, and much less frequently in non-pandemic HIV-1 groups O, N and P. In addition, the study found a statistically significant positive correlation between the frequency with which the ASP ORF was present in each HIV-1 subtype and the prevalence of the subtype, suggesting that ASP might be involved in virus spread. The authors also noted that, while 16% of HIV-1 subtypes A, B, C, G and CRF01_AE lack the ASP ORF, they also lack the *nef* gene at a similar frequency [77].

In the same report, Cassan and colleagues performed computer simulations to estimate the likelihood that the presence of ASP in 77% of HIV-1 group M strains was due to chance [71]. They found that the likelihood of finding a 180-codon ORF in the -2 frame of a gene the same size as *env* or of a genome the same size as HXB2 were 3% and 19%, respectively—much lower than the actual frequency of group M sequences with the ASP ORF. They concluded that the presence of ASP at such high frequency was unlikely due to mere chance [71].

The evolution and selective pressure of *asp* were investigated in three different studies. The first report is the same study by Cassan et al. discussed above [71]. These authors found that the canonical *start* codon of the ASP ORF is conserved in 97% of the HIV-1 group M sequences. However, conservation of the ASP *start* codon at such high frequency was not imposed by the sequence of *env* on the opposite strand, because point mutations are possible that would cause loss of the *start* codon for *asp* while producing a synonymous codon in *env* [71]. The authors made a similar observation in regard to *stop* codons: there are 11 sites along the ASP ORF that could potentially mutate and create a *stop* without affecting the amino acid sequence of ENV. While seven of these sites are actual *stop* codons in sequences of HIV-1 groups N, O and P, they are *stop*s in only 0.5% of the group M sequences. Moreover, all sequences belonging to HIV-1 group M subtype A contain an early *stop* codon located 12 codons downstream of the *start* codon in *asp*. However, 90% of these sequences contain an alternative *start* codon located 17 codons downstream of the early *stop* codon [71]. Therefore, these results show a selective pressure to conserve an intact ASP ORF by maintaining a *start* codon (or by creating it a new one when lost, as in the case of subtype A sequences), and by avoiding early *stop* codons [71].

Two subsequent reports analyzed co-evolution of the ASP and ENV open reading frames. Dimonte studied the correlation between mutations in ASP and ENV, and CCR5 vs. CXCR4 coreceptor usage [78]. The author first used the G2P algorithm to predict the tropism of ~24,000 HIV-1 subtype B *env* sequences deposited in the Los Alamos HIV Sequence Database, and then analyzed the association of mutations in ASP with predicted coreceptor usage. He found that mutations in 36 amino acids of ASP are associated with preferential CCR5 or CXCR4 usage. A number of these mutations localize in the loop between the two transmembrane domains of ASP, which we have shown to be exposed in the extracellular or ‘extra-viral’ environment, thus suggesting a possible involvement of ASP in cell entry [57]. Next, Dimonte investigated the association between mutations in ASP and in the V3 loop of gp120, and coreceptor usage. The gp120 V3 loop becomes exposed upon binding of gp120 with CD4, contacts the coreceptor (CCR5 or CXCR4), and mediated virus entry into the target cell [79]. The author found eight sites on ASP where specific mutations are associated in a statistically significant manner with mutations in the V3 loop of gp120 and with CCR5/CXCR4 tropism; six of these eight mutations map in the extracellular/viral loop of ASP [78]. It should be underscored that the ASP ORF overlaps the *env* gene in the region of the V4 and V5 loops, but not V3. Therefore, association of specific residues in ASP and in the V3 loops of ENV is not genetically linked, but possibly functionally linked.

Finally, a recent report used a new computer algorithm called OLGenie to study co-evolution of the *env* and *asp* genes [80]. The ratio of nonsynonymous and synonymous mutations (i.e., nucleotide substitutions that alter or do not alter, respectively, the amino acid sequence of the protein product) within a gene is represented as *d*_N_/*d*_S_, and is an indicator of selective pressure. In protein-coding genes, synonymous mutations typically exceed nonsynonymous mutations (*d*_N_/*d*_S_ < 1), which are often deleterious (especially in functionally-constrained regions of the gene), and therefore are removed by negative or ‘purifying’ selection. However, when nonsynonymous mutations provide a selective advantage, they will become fixed in the population by positive selection. In the special case of overlapping genes—such as in the case of *env* and *asp*—synonymous mutations in one of the two ORFs could be nonsynonymous in the other, and this may impact the *d*_N_/*d*_S_ value. The OLGenie algorithm was developed to study evolution of overlapping genes [80]. When applied to study *asp*, OLGenie found that this gene is under intense purifying selection (*d*_N_/*d*_S_ = 0.29). This study strongly suggests that mutations occurring within the ASP ORF are disproportionately synonymous, indicating an evolutionary effort to maintain the amino acid sequence of ASP [80].

Altogether, the studies described above demonstrate that the ASP ORF is present almost exclusively in pandemic HIV-1 subtypes, and that the percentage of viral strains with a full-length ASP within each HIV-1 subtype correlates with the worldwide prevalence of the subtype. It also appears that a significant evolutionary effort has been invested toward the maintenance of an intact ORF (preservation of *start* codon and avoidance of *stop* codons) and the conservation of the amino acid sequence (low *d*_N_/*d*_S_ ratio). Finally, there is evidence of a correlation between certain ASP variants and viral tropism. When considering the small size and the complexity of the HIV-1 genome, and the intense immune pressure acting on the virus, it would seem highly unlikely that the *asp* gene appeared by chance, and that it would be conserved if it did not encode for a protein product, or that such a protein did not provide any selective advantage to the virus.

## 8. Concluding Remarks

Since its identification more than three decades ago, there have been very few studies on the antisense protein of HIV-1. That is surprising when considering the strong association between the presence of a full-length ASP ORF and HIV-1 subtype prevalence, and also how conserved the ASP sequence is among pandemic HIV-1 strains. Since the antisense protein HBZ has been proven to play a crucial role in the pathogenesis of HTLV-1, it is equally reasonable to hypothesize that its HIV-1 counterpart, ASP, plays an important role in the HIV-1 pandemic.

The role of ASP in HIV-1 replication, spread and pathogenesis remains largely unknown. However, the fact that the ASP ORF is present only in pandemic HIV-1 strains, and that its conservation and length correlate with subtype prevalence [71], along with the evidence that the *asp* gene presents synonymous mutations at a disproportionately high rate [80] strongly suggest that ASP is involved in the virus life cycle. Studies from our and other groups have begun to make some progress in that direction, and these lines of investigation are likely to continue and to produce valuable information. Achieving a complete understanding of the role that ASP has played in inter- and intra-species spread, and that it continues to play in intra-host spread will require new efforts, new research tools, and—most importantly—new and original hypotheses.

## Figures and Tables

**Table 1:** Evidence of ASP expression in HIV-1 infected individuals.

Target	Samples	Assay	Findings	Refs.
Anti-ASP antibodies	Sera from HIV-1 patients	IP	Sera from 15 HIV-1 patients were able to immunoprecipitate in vitro translated ASP; patients were at stage I, III and IV of infection during pre-HAART era	[62]
		LIPS	Antibodies to ASP are detectable in serum of untreated patients, but not in serum of treated patients or in serum of HIV-1 controllers	[63]
CTL responses against ASP	PBMC; acute and chronic; on and off HAART	ELISpot	PBMC from chronically infected patients both on and off HAART reacted to peptide pools spanning the ASP open reading frame	[64]
	PBMC; on and off HAART	ELISpot ICS	Detection of CD8+ T cell responses against ASP in patients off HAART; CD8+ T cells responses included production of cytokines and chemokines	[65]
	PBMC; chronic patients	ELISpot	Detection of CTL responses against several peptides matching the ASP consensus sequence	[66]
Antisense RNA	CD4+ T cells from treated patients	Strand-specific RT-qPCR	10–30 copies of HIV-1 antisense RNA per 10^6^ resting CD4+ T cells without in vitro activation	[67]
	CD4+ T cells from treated and untreated patients	RT-qPCR with biotinylated RT primer	After anti-CD3/CD28 activation for 5 days: 5–10 copies of antisense RNA per 10^6^ cells from treated patients; 20–2 × 10^6^ copies of antisense RNA per 10^6^ cells from untreated patients	[68]

## Data Availability

No new data were created or analyzed in this study. Data sharing is not applicable to this article.
